# Effect of Biochar and Well-Rotted Manure on Maize Yield in Intercropping Systems Based on High-Throughput Sequencing Technology

**DOI:** 10.3390/plants14243696

**Published:** 2025-12-05

**Authors:** Hui Liu, Wenlong Zhang, Wanyu Dou, Yutao Li, Guoxin Shi, Wei Pei

**Affiliations:** 1 College of Arts and Science, Northeast Agricultural University, Harbin 150030, China; liuhui@neau.edu.cn; 2 College of Engineering, Northeast Agricultural University, Harbin 150030, China; s230701050@neau.edu.cn (W.Z.); 13946232354@163.com (W.D.); 3 Heilongjiang River Fisheries Research Institute, Chinese Academy of Fishery Sciences, Harbin 150030, China; liyutao@neau.edu.cn; 4 College of Agriculture, Northeast Agricultural University, Harbin 150030, China; shiguoxin0318@163.com

**Keywords:** intercropping, biochar, manure, nitrogen cycling, maize yield

## Abstract

Biochar and well-rotted manure are commonly employed materials for sustainable agricultural development, possessing the potential to consistently enhance the yield of monoculture crops. However, their impact on the stability of crop yields in intercropping systems, as well as the microenvironment of the border-row rhizosphere, remains inadequately understood. Consequently, this study utilized corn stover biochar and well-rotted pig manure while minimizing the application of chemical fertilizers to investigate the synergistic effects of biochar and composted manure in augmenting maize yield within a soybean–maize intercropping system and regulating the nitrogen cycle in the border-row rhizosphere under reduced fertilization conditions. In comparison to traditional fertilization, the combination of biochar and manure under reduced fertilization conditions significantly increased the contents of ammonium nitrogen (55%), dissolved organic nitrogen (523%), and particulate organic nitrogen (833%) while simultaneously decreasing the content of mineral-associated organic nitrogen (60%). Additionally, this combination synergistically reduced urease activity (22%) while enhancing the activities of nitrogenase (11%), nitrate reductase (297%), and hydroxylamine reductase (20%). This study establishes a theoretical foundation for elucidating how organically amended materials consistently enhance productivity in intercropping systems and alter nitrogen ecology in border-row rhizospheres, offering new perspectives on sustainable fertilization strategies and crop patterns.

## 1. Introduction

Maize is a globally important food and energy crop [1]. Increasing corn yields has become a key measure for addressing climate change and ensuring food security [2]. In recent years, maize–soybean intercropping has been shown to sustainably enhance soil fertility and maize yield and biomass [3,4]. However, crop yields in this pattern vary significantly, primarily constrained by soil nutrient availability, particularly nitrogen [5,6]. Consequently, optimizing intercropped maize productivity under low-fertilizer conditions has emerged as a critical research priority in sustainable agronomy [7]. Although extensive literature has investigated nutrient cycling, including nitrogen cycling [8], as well as root interaction mechanisms in the belowground components of intercropping systems, current research predominantly focuses on optimizing aboveground field configurations, such as row ratios and strip widths or ecological niche advantages, to achieve stable yield enhancement in intercropping systems [9]. Limited attention has been given to exploring organic amendments such as biochar and manure for improving intercropped yields. Therefore, this study, based on a reduction in chemical fertilizer usage, verifies that the combined application of biochar and manure can stably enhance the yield of intercropped crops, and it confirms that the increase in crop yield is closely related to the nitrogen dynamics in the rhizosphere of border rows.

Currently, studies have demonstrated that amendments with biochar and organic fertilizers improve nitrogen cycling and the functional microbial network within rhizosphere ecology [10]. However, research on the impact of biochar on the mutual transformation of nitrogen components in the rhizosphere remains inconsistent [11,12], with effects becoming more complex when combined with organic fertilizers [13,14]. For instance, while biochar typically reduces the content of inorganic nitrogen in the soil, its co-application with manure increases the soil’s inorganic nitrogen content [15]. Biochar can absorb nitrification inhibitors (such as phenolic substances) from the soil, thereby activating nitrifying microorganisms to promote nitrification, while manure further enhances nitrification by supplying organic matter [16,17]. The application of biochar and organic fertilizers in intercropping systems further complicates these dynamics. As a significant component of soil, microorganisms play a crucial role in elucidating the synergistic promotion of boundary root nitrogen dynamics by biochar and organic fertilizers. They are involved in regulating multiple nitrogen metabolic pathways, including nitrogen fixation, assimilation, ammonification, anammox, nitrification, and denitrification [18]. However, a substantial body of research has shown that intercropping can drive changes in the composition of rhizosphere microbial communities [19,20], yet knowledge regarding the effects of biochar and organic fertilizers on microbial-mediated nitrogen transformation under intercropping conditions remains limited.

To address these challenges, a field experiment focusing on the rhizosphere of border rows in maize–soybean strips was conducted, examining how crop yield is affected by combinations of biochar and well-rotted manure under reduced fertilization conditions. It was hypothesized that biochar and composted manure, through synergistic regulation of bacterial and fungal functional groups closely related to the nitrogen cycle, alter the nitrogen dynamics and nitrogen-transforming enzyme activities in the rhizosphere of border rows, thereby consistently enhancing the yield of maize crops in intercropping systems under reduced fertilization conditions. The objectives of this experiment are (1) to explore the correlation between the synergistic effects of biochar and composted manure on improving maize crop yield and the nitrogen dynamics in the rhizosphere of border rows under reduced fertilization conditions; (2) to elucidate the species composition and community distribution of nitrogen-functional microbial groups in the rhizosphere of border rows under the synergistic effects of biochar and composted manure, as well as the network relationships among these communities; and (3) to identify the key bacterial and fungal species responsible for differences in nitrogen functionality in the rhizosphere of border rows and to determine the response mechanisms of microbial-mediated nitrogen dynamics and nitrogen-transforming enzyme activities to the application of biochar and organic manure.

## 2. Results

### 2.1. Maize Yield in Intercropping Systems

**Figure 1:**
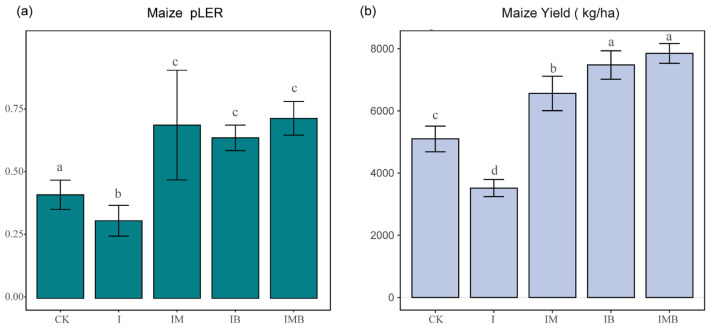
Analysis of maize crop yield per unit area and partial land equivalent ratio in the intercropping system. (**a**) Partial land equivalent ratio analysis of maize crop yield. (**b**) Analysis of yield differences in intercropped maize under different treatments. CK, I, IM, IB, and IMB denote the conventional level of inorganic fertilizer (control), 30% reduction in inorganic fertilizer, 30% reduction in inorganic fertilizer mixed with manure, 30% reduction in inorganic fertilizer mixed with biochar, and 30% reduction in inorganic fertilizer mixed with biochar and manure, respectively. Different lowercase letters represent significant differences (0.05) between treatments.

The land equivalent ratio (LER) is used to measure the land use efficiency of intercropping compared to monoculture in agricultural production. An LER > 1 indicates that intercropping has a per-unit-area advantage over monoculture in improving resource utilization [21]. Under reduced fertilizer conditions, the addition of biochar and mature manure not only significantly improved the land use efficiency but also greatly increased the maize crop yield within the intercropping system (Figure 1). Specifically, compared with the CK treatment group, the partial land equivalent ratio (pLER) of the I treatment group noticeably decreased (*p* < 0.05; Figure 1a), indicating that simply reducing the fertilization rate by 30% reduces soil fertility while also decreasing land use efficiency. Meanwhile, the pLERs of IB, IM, and IMB noticeably increased (*p* < 0.05; Figure 1a), demonstrating that biochar and mature manure significantly enhance land use efficiency under reduced fertilization conditions. Additionally, compared with CK, I led to a significant 31% decrease in maize crop yield (*p* < 0.05; Figure 1b). However, compared with the I treatment, the IM, IB, and IMB treatments resulted in maize crop yield significantly increasing by 87%, 114%, and 124%, respectively (*p* < 0.05; Figure 1b), demonstrating that with reduced fertilizer, biochar and mature manure effectively provided additional yield enhancement for maize. More importantly, compared with the CK treatment, the IM, IB, and IMB treatments resulted in a significant increase in maize crop yield by 28%, 47%, and 54%, respectively (*p* < 0.05; Figure 1b). This indicates that applying biochar and manure has a significant compensatory effect on the yield reduction caused by reducing fertilizer.

### 2.2. Composition of the Rhizosphere Nitrogen Pool

**Figure 2:**
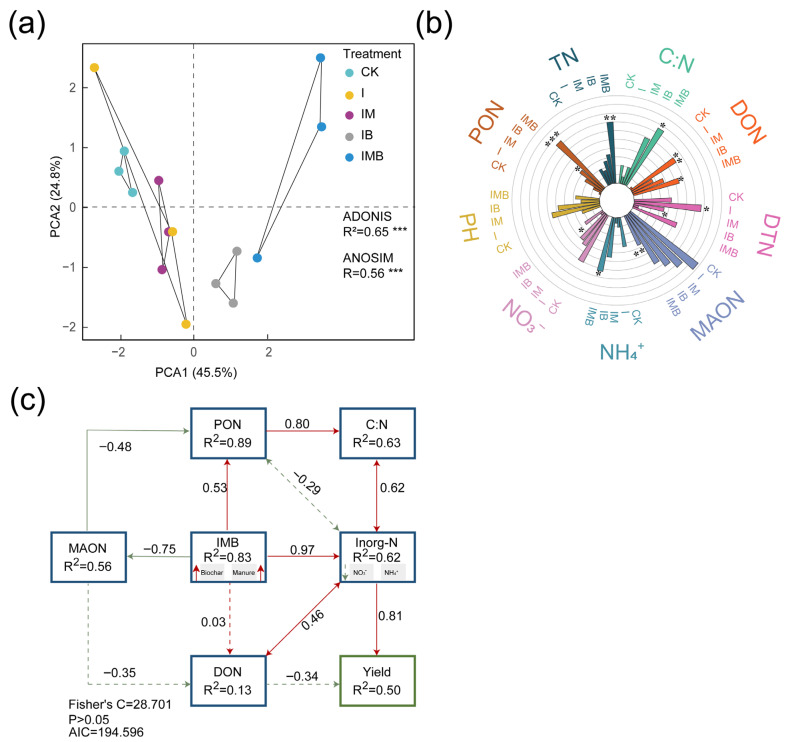
Analysis of nitrogen pool components in rhizosphere soil under different treatments. (**a**) PCA: colors represent treatment groups. (**b**) Analysis of differences in soil nitrogen components under different fertilization treatments. (**c**) The segmented structural equation model, with the numbers near the arrows representing standardized path coefficients, uses solid lines to indicate significance and dashed lines to indicate non-significance. Double-headed arrows denote covariances between variables, while single-headed arrows represent directional relationships. CK, I, IM, IB, and IMB denote the conventional level of inorganic fertilizer (control), 30% reduction in inorganic fertilizer, 30% reduction in inorganic fertilizer mixed with manure, 30% reduction in inorganic fertilizer mixed with biochar, and 30% reduction in inorganic fertilizer mixed with biochar and manure, respectively. Different lowercase letters represent significant differences (0.05) between treatments. * *p* < 0.05, ** *p* < 0.001, *** *p* < 0.0001.

Principal component analysis (PCA) revealed significant differences in nitrogen components in the rhizosphere of border rows under different fertilization treatments (*p* < 0.001; Figure 2a). The greater the degree of separation of each sample in the figure, the more pronounced the classification effect between treatment groups and the more reliable the grouping factor. The IB, IM, and IMB treatments were distinctly separated from the CK treatment along the PCA1 axis (Figure 2a). This indicates that biochar and composted manure synergistically significantly altered the spatial distribution structure of nitrogen components.

Compared to CK, the I treatment resulted in a significant increase of 590% and 23% in the content of dissolved organic nitrogen (DON) and dissolved total nitrogen (DTN), respectively (*p* < 0.05; Figure 2b), while the content of ammonium nitrogen (NH_4_^+^-N) and nitrate nitrogen (NO_3_^−^-N) decreased by 42% and 18%, respectively (*p* > 0.05; Figure 2b). This indicates that reducing inorganic fertilizer application resulted in a significant increase in the concentration of freely dissolved small-molecule organic nitrogen. Compared to the I treatment, IB significantly reduced the DTN by 39% (*p* < 0.05; Table S3) but significantly increased the NH_4_^+^-N content by 112% (*p* < 0.05; Table S3). However, compared to CK, IB significantly increased the content of particulate organic nitrogen (PON) by 360% (*p* < 0.05; Figure 2b) and significantly decreased the NO_3_^−^-N content by 59% (*p* < 0.05; Figure 2b). Additionally, compared to the I treatment, IM significantly decreased the DTN content by 23% (*p* < 0.05; Table S3). However, compared with CK, IM showed no significant changes in various nitrogen components (*p* > 0.05; Figure 2b), indicating that under reduction conditions, the application of manure maintained nitrogen pool stability comparably to conventional fertilization. Furthermore, compared with the I treatment, IMB significantly increased the PON content and DTN content by 103% and 43%, respectively (*p* < 0.05; Table S3), demonstrating that under reduced fertilizer conditions, the combination of biochar and matured manure synergistically enhanced the accumulation of organic nitrogen. Compared to CK, IMB significantly increased the contents of PON, NH_4_^+^-N, and DON by 833%, 55%, and 523%, respectively (*p* < 0.05; Figure 2b). The content of mineral-associated organic nitrogen (MAON) was significantly reduced by 60% (*p* < 0.05; Figure 2b), indicating that under reduction conditions, the application of biochar and matured manure promoted the conversion of sequestered organic nitrogen into short-term available nitrogen.

Furthermore, a structural equation model (SEM) was established to analyze the nitrogen components and maize yield (Figure 2c). This model revealed the relationship between biochar and matured manure in regulating the conversion of organic nitrogen–inorganic nitrogen pools and yield under reduction conditions. Biochar and matured manure had the most significant direct positive impact on the inorganic nitrogen pool. By reducing the MAON element and increasing the PON element, they enhanced the C:N ratio, thereby positively affecting the inorganic nitrogen pool and indirectly increasing maize yield. Additionally, the model showed consistent effects of biochar and matured manure on nitrogen content and yield. This indicates that biochar and mature manure jointly either directly affect the inorganic nitrogen pool or indirectly affect it by altering the C:N ratio, leading to increased maize yield.

### 2.3. Rhizosphere Potential Activity of Nitrogen-Transforming Enzymes

**Figure 3:**
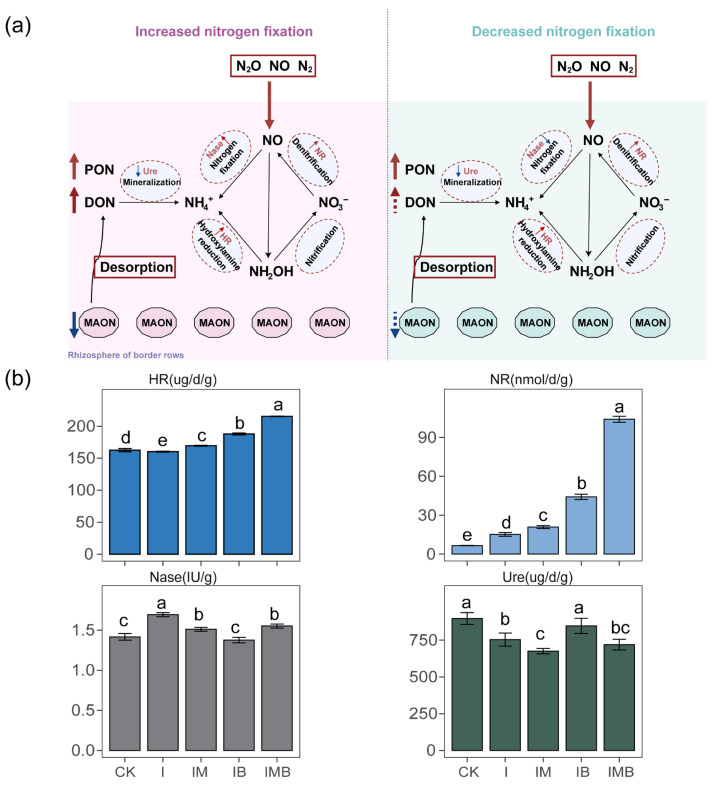
Analysis of rhizosphere soil enzymes under different treatments. (**a**) Dynamic analysis of enzymes within the intercropping system, with the red area representing the IMB treatment group and the blue area representing the IB treatment group. The enzymes required for this turnover process are marked on the arrows. (**b**) Analysis of differences in rhizosphere soil enzymes under different treatments. CK, I, IM, IB, and IMB denote the conventional level of inorganic fertilizer (control), 30% reduction in inorganic fertilizer, 30% reduction in inorganic fertilizer mixed with manure, 30% reduction in inorganic fertilizer mixed with biochar, and 30% reduction in inorganic fertilizer mixed with biochar and manure, respectively. Different lowercase letters represent significant differences (0.05) between treatments.

Under the reduction conditions, the application of biochar and mature manure significantly altered the potential activity of nitrogen transformation enzymes (*p* < 0.05; Figure 3a). Compared to CK, the I treatment significantly increased the nitrate reductase and nitrogenase contents by 131% and 19%, respectively (*p* < 0.05; Figure 3b), while significantly decreasing the urease content by 16% (*p* < 0.05; Figure 3b). Compared to the I treatment, IB significantly increased the hydroxylamine reductase, nitrate reductase, and urease contents by 17%, 191%, and 12%, respectively, while reducing nitrogenase content by 18% (*p* < 0.05; Figure 3b). However, compared with CK, IB showed a significant increase of 574% and 16% in nitrate reductase and hydroxylamine reductase contents, respectively (*p* < 0.05; Figure 3b). Furthermore, compared to the I treatment, IM resulted in a significant increase of 6% and 218% in hydroxylamine reductase and nitrate reductase contents, respectively (*p* < 0.05; Figure 3b), as well as a significant decrease of 25% and 6% in urease and nitrogenase contents, respectively (*p* < 0.05; Figure 3b). This indicates that under reduced fertilizer conditions, the application of mature manure promoted the denitrification process, while simultaneously inhibiting biological nitrogen fixation and the mineralization process of urea nitrogen. Furthermore, compared to the I treatment, IMB significantly increased hydroxylamine reductase and nitrate reductase contents by 34% and 585%, respectively (*p* < 0.05; Figure 3b), while significantly decreasing nitrogenase content by 8% (*p* < 0.05; Figure 3b). This indicates that under reduced fertilizer conditions, the synergy of biochar and mature manure similarly enhanced the denitrification process but also inhibited the biological nitrogen fixation process. However, compared to CK, IMB significantly reduced the urease content by 20% (*p* < 0.05; Figure 3b), while significantly increasing the activities of nitrate reductase, hydroxylamine reductase, and nitrogenase by 1487%, 32%, and 9%, respectively (*p* < 0.05; Figure 3b). This indicates that under the reduction conditions, the application of biochar and mature manure enhanced the biological nitrogen fixation and denitrification processes but reduced the dependence on urea nitrogen mineralization (Figure 3a).

### 2.4. Rhizosphere Microbial Structure Composition and Community Distribution

**Figure 4:**
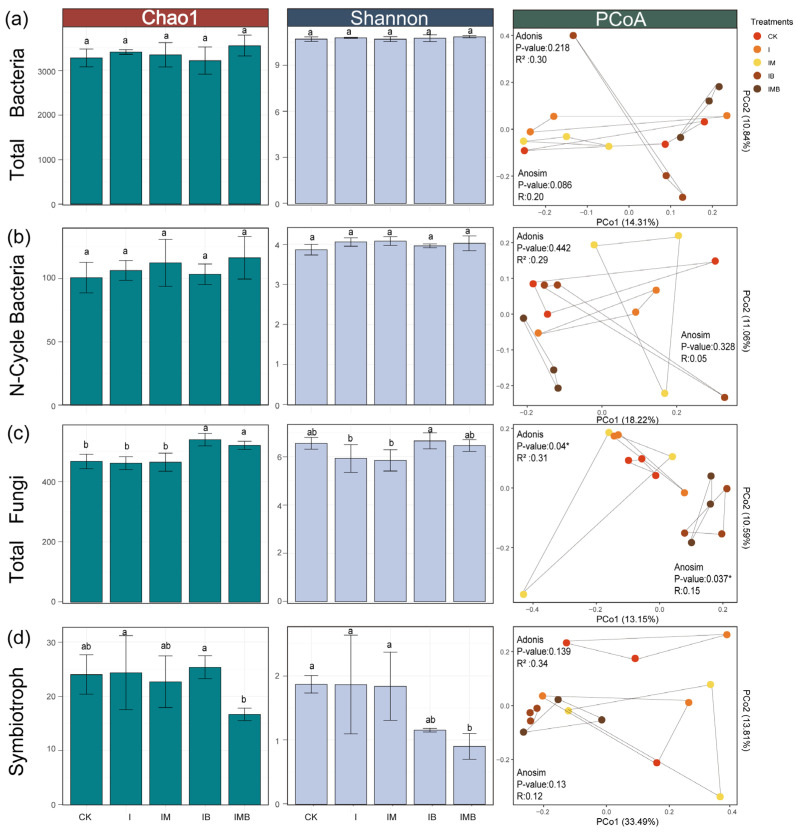
Diversity analysis of rhizosphere microbial communities. (**a**) The Chao1 index, Shannon index, and PCoA analysis of total bacteria. (**b**) The Chao1 index, Shannon index, and PCoA analysis of N-cycle bacteria. (**c**) The Chao1 index, Shannon index, and PCoA analysis of total fungi. (**d**) The Chao1 index, Shannon index, and PCoA analysis of symbiotroph. All PCoA analyses based on Jaccard distance, with colors representing different treatment groups. CK, I, IM, IB, and IMB denote the conventional level of inorganic fertilizer (control), 30% reduction in inorganic fertilizer, 30% reduction in inorganic fertilizer mixed with manure, 30% reduction in inorganic fertilizer mixed with biochar, and 30% reduction in inorganic fertilizer mixed with biochar and manure, respectively. Different lowercase letters represent significant differences (0.05) between treatments. * *p *< 0.05.

Compared with CK, there were no significant changes in the Chao1 and Shannon indices of bacterial and fungal communities under the I treatment (*p* > 0.05; Figure 4a,c), indicating that reducing the amount of fertilizer had no significant impact on the species richness and evenness of bacterial and fungal communities. The Chao1 index of the fungal community under the IB and the IMB treatments increased significantly (*p *< 0.05; Figure 4a), while the Shannon index showed no significant change (*p* > 0.05; Figure 4c), indicating that under reduced fertilizer conditions, the addition of biochar and composted manure increased the total number of species in the fungal community but did not lead to a change in the evenness of the community. The unconstrained PCoA analysis revealed that each treatment had a significant impact on the distribution characteristics of fungal community structures (*p *< 0.05; Figure 4c). Specifically, the fungal communities of the IB, IMB, and CK groups were distinctly separated along axis 1 (Figure 4c), indicating a re-niche differentiation of the fungal communities. Combined with the diversity analysis results, it was demonstrated that under reduction conditions, biochar treatment or the combination of biochar and composted manure treatment altered the microenvironment of the border-row rhizosphere, attracting or activating the growth of certain low-abundance or function-specific fungal taxa, while the impact on dominant fungi might be relatively minor.

According to their functional classification, compared with the CK treatment, the I treatment did not significantly alter the Shannon index or Chao1 index of nitrogen-cycling bacteria and symbiotic fungi (*p* > 0.05; Figure 4b,d), indicating that reducing the fertilization rate did not significantly change the total species number and evenness of nitrogen-cycling bacteria and symbiotic fungi in the border-row rhizosphere. The IMB treatment significantly decreased the Shannon index of symbiotic fungi (*p *< 0.05; Figure 4d), but there was no significant change in the Chao1 index, suggesting that under reduced fertilization conditions, the addition of biochar and matured manure decreased the evenness of symbiotic fungi, rather than the overall species number. In addition, the combined application of biochar and matured manure, as well as their individual applications, did not significantly affect the species structure composition and community distribution of nitrogen-cycling bacteria in the rhizosphere of border rows. The above results indicate that under reduced fertilizer conditions, compared to bacterial communities, the biochar and matured manure treatment groups have a greater impact on the total number of species and evenness of fungal communities.

**Figure 5:**
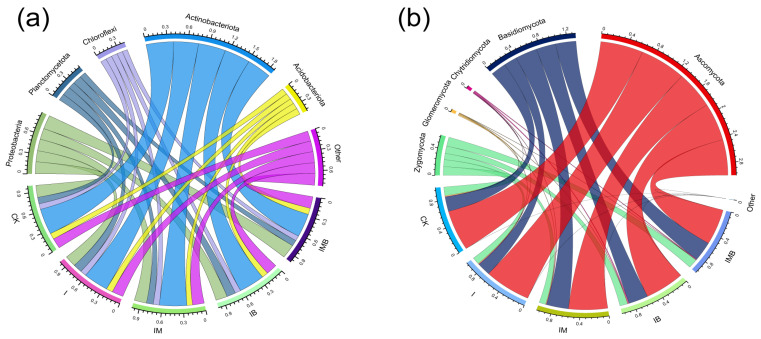
Microbial species composition (TOP5) at the phylum level in rhizosphere soil. (**a**) Bacterial species composition at the phylum level. (**b**) Fungal species composition at the phylum level. CK, I, IM, IB, and IMB denote the conventional level of inorganic fertilizer (control), 30% reduction in inorganic fertilizer, 30% reduction in inorganic fertilizer mixed with manure, 30% reduction in inorganic fertilizer mixed with biochar, and 30% reduction in inorganic fertilizer mixed with biochar and manure, respectively. Different colors represent different treatment groups.

Based on their relative abundance, *Actinobacteriota* (35–39%), *Proteobacteria* (17–21%), *Acidobacteriota* (8–9%), *Planctomycetota* (9–13%), and *Chloroflexi* (8–9%) are the dominant bacterial phyla in the rhizosphere of border rows (Figure 5a). Among these, the phyla *Actinobacteriota*, *Proteobacteria*, and *Planctomycetota* contain branches of genera involved in nitrogen-cycling functions. Additionally, within the branches of non-dominant microbial groups such as *Cyanobacteria*, *Nitrospirota*, and *Verrucomicrobiota*, there are also genera involved in the nitrogen cycle. Compared with CK, IMB significantly increased the relative abundance of *Verrucomicrobiota*, *Fibrobacterota*, and *Tenericutes* phyla and significantly decreased the relative abundance of the *Nitrospirota* phylum (*p *< 0.05; Figure S1a). This indicates that under reduced fertilizer conditions, the synergistic effect of biochar and mature manure altered the relative abundance of low-abundance nitrogen-cycling functional groups, while having a lesser impact on high-abundance nitrogen functional groups. Additionally, *Ascomycota* (55–68%), *Basidiomycota* (20–32%), *Zygomycota* (10–14%), *Glomeromycota* (1%), and *Chytridiomycota* (1%) are the dominant fungal groups in the rhizosphere of border rows (Figure 5b). Among these, *Ascomycota* and *Glomeromycota* are symbiotic fungal groups, closely associated with the nitrogen cycle. Compared with CK, IMB significantly reduced the relative abundance of *Glomeromycota* (*p *< 0.05; Figure S1b), indicating that under reduction fertilizer conditions, biochar and matured manure decreased the relative abundance of *Glomeromycota*, the sole taxonomic group of arbuscular mycorrhizal fungi.

### 2.5. Rhizosphere Nitrogen-Functional Microbial Networks

**Figure 6:**
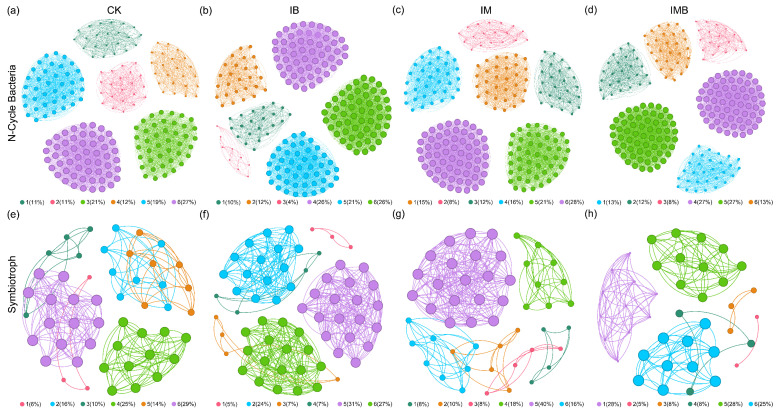
Co-occurrence network of rhizosphere soil nitrogen-cycling bacteria and symbiotic fungal communities. (**a**–**d**) are the network diagrams of nitrogen-cycling bacteria under different treatments. (**e**–**h**) are the network diagrams of symbiotic fungi under different treatments. Different colors represent different modules. CK, I, IM, IB, and IMB denote the conventional level of inorganic fertilizer (control), 30% reduction in inorganic fertilizer, 30% reduction in inorganic fertilizer mixed with manure, 30% reduction in inorganic fertilizer mixed with biochar, and 30% reduction in inorganic fertilizer mixed with biochar and manure, respectively.

To further investigate the responses of nitrogen-cycling bacteria and symbiotic fungi niches in the rhizosphere of boundary rows to biochar and composted manure treatments, nitrogen-cycling bacterial networks and symbiotic fungal networks under different fertilization treatments were constructed (Figure 6). These eight networks exhibited significant differences in their structure and topology (Tables S7–S9), where the degree distribution and clustering coefficient reflected the connectivity among nodes in the networks [22]. Compared to CK, IB increased the number of edges (4531), the number of nodes (211), and the average degree (42.95) in the nitrogen cycle bacterial network but decreased the proportion of positive links (88%) (Figure 6b, Table S7), indicating that biochar enhanced the growth, complexity, and connectivity of the interaction network of nitrogen cycle bacterial communities under reduced fertilizer conditions. IM increased the number of edges (216), the number of nodes (4375), and the average degree (40.51) in the nitrogen cycle bacterial network (Figure 6c, Table S7), demonstrating that matured manure also increased the complexity and connectivity of the nitrogen cycle bacterial network under reduction conditions. IMB increased the numbers of edges (5030) and nodes (226) and the average degree (44.51) in the nitrogen-cycling bacterial network, but reduced the proportion of positive connections (87%) (Figure 6d, Table S7), indicating that biochar and matured manure enhanced competition among nitrogen-cycling bacterial communities under the reduction conditions. Additionally, IB increased the number of edges (427) and nodes (62), the average degree (13.77), and the proportion of positive links (98%) in the symbiotic fungal network (Figure 6f, Table S8), suggesting that biochar promoted cooperation and nutritional complementarity among symbiotic fungal communities under reduced fertilizer conditions. IM increased the number of edges (276) and nodes (50) and the average degree (11.04) in the symbiotic fungal network (Figure 6g, Table S8), indicating that composted manure enhanced the complexity and connectivity of the symbiotic fungal network under reduced nitrogen conditions. IMB reduced the number of edges (162) and nodes (40) and the average degree (8.10) but increased the proportion of positive connections (98%) (Figure 6h, Table S8), suggesting that biochar and composted manure decreased the complexity and connectivity of the symbiotic fungal network under reduction conditions while simultaneously enhancing cooperation.

To further reveal the key species in the nitrogen cycle bacterial network and symbiotic fungal network characteristics, LefSe analysis was used to screen out the core species (Figures S2 and S3), and cross-validation was performed using a random forest (Figure S4). Among them, the core species of the nitrogen cycle bacterial network were *Nitrosospira*, *Opitutus*, *Mesorhizobium*, *Klebsiella*, *Anaerostipes*, *Starkeya*, *Klebsiella*, *Rhizobacter*, *Singulisphaera*, *Schlesneria*, and *Herbaspirillum* (Figure S2). The LefSe results showed that, compared to CK, IMB significantly increased the relative abundance of the genera *Mesorhizobium*, *Opitutus*, *Nitrosospira*, *Anaerostipes*, and *Starkeya* in the rhizosphere of the boundary row and significantly decreased the relative abundance of the genus *Klebsiella* (*p *< 0.05; Figure S2a). Additionally, the core species of the symbiotic fungal network was the genus *Rhizophagus* (Figure S3). Compared to CK, IMB significantly reduced the relative abundance of the genus *Rhizophagus* (*p *< 0.05; Figure S3a). The random forest also revealed that the genera *Mesorhizobium*, *Opitutus*, *Nitrosospira*, *Anaerostipes*, and *Starkeya* were significantly enriched in the rhizosphere of IMB, while the genera *Klebsiella* and *Rhizophagus* were significantly reduced (Figure S4).

### 2.6. Correlation Analysis of Nitrogen Components, Nitrogen Metabolism Enzymes, and Nitrogen-Functional Microorganisms in the Rhizosphere

**Figure 7:**
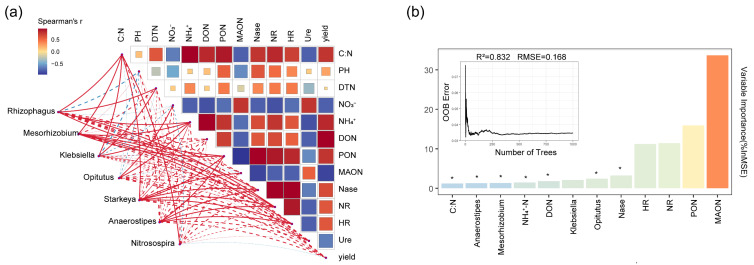
Analysis of the relationships between environmental factors, nitrogen-cycling bacteria, and symbiotic fungal species in the IMB rhizosphere. (**a**) Mantel test analysis of the relationships between species at the genus level and environmental factors. Red indicates positive correlations, green indicates negative correlations, solid lines represent significant correlations, and dashed lines represent non-significant correlations. (**b**) Ranking of the importance of environmental factors and core genera predicted by the random forest regression model. * *p *< 0.05.

To further reveal the effects of biochar and composted manure treatments on the microenvironment and inter-species relationships of functional species in the border-row rhizosphere, the Mantel test analysis tool was used to analyze the correlation between environmental factors and core species (Figure 7a). The Mantel test analysis shows that in the rhizosphere treated with biochar and composted manure, the genus *Klebsiella* showed a significant positive correlation with the content of particulate organic nitrogen (*p* < 0.05; Figure 7a). The genus *Mesorhizobium* exhibited significant positive correlations with the contents of ammonium nitrogen, particulate organic nitrogen, and nitrate reductase, respectively (*p* < 0.05; Figure 7a). The genus *Opitutus* showed significant positive correlations with the contents of nitrate nitrogen and ammonium nitrogen, respectively (*p* < 0.05; Figure 7a). The genus *Starkeya* showed significant positive correlations with the contents of particulate organic nitrogen, mineral-associated organic nitrogen, nitrogenase, nitrate reductase, and hydroxylamine reductase (*p* < 0.05; Figure 7a), indicating that under low-fertilizer conditions, the nitrogen dynamics and metabolic enzyme activities in the rhizosphere treated with biochar and matured manure were influenced by the core functional species. The genus *Rhizophagus* exhibited significant positive correlations with the contents of ammonium nitrogen, particulate organic nitrogen, nitrate reductase, and hydroxylamine reductase, respectively (*p* < 0.05; Figure 7a). Additionally, in the rhizosphere under biochar and matured manure treatments, the genera *Rhizophagus*, *Mesorhizobium*, *Klebsiella*, *Starkeya*, and *Nitrosospira* were all significantly positively correlated with the C:N ratio (*p* < 0.05; Figure 7a), indicating that under reduction fertilizer conditions, the core functional species treated with biochar and matured manure are influenced by the C:N ratio. Furthermore, when treated with biochar and composted manure, the urease activity was significantly positively correlated with nitrate nitrogen content and mineral-associated organic nitrogen content (*p* < 0.05; Figure 7a) and significantly negatively correlated with ammonium nitrogen (*p* < 0.05; Figure 7a). This indicates that when treated with biochar and composted manure, the increase in ammonium nitrogen concentration reduced the reliance on exogenous urea nitrogen, thereby weakening urease activity. Additionally, under treatments with biochar and mature manure, the activity of rhizosphere nitrogenase was significantly positively correlated with the contents of ammonium nitrogen, soluble organic nitrogen, and particulate organic nitrogen (*p* < 0.05; Figure 7a), while it was significantly negatively correlated with the contents of nitrate nitrogen and mineral-associated organic nitrogen (*p* < 0.05; Figure 7a). Moreover, it was also significantly negatively correlated with urease activity (*p* < 0.05; Figure 7a). These results indicate that under reduced fertilizer conditions, biochar and mature manure increased the concentration of ammonium nitrogen by enhancing the activity of nitrogenase, thereby reducing the reliance on exogenous urea nitrogen. This, in turn, weakened urease activity, ensured the accumulation of organic nitrogen, and enhanced the nitrogen fixation. Additionally, a random forest regression model was constructed to assess the impact of various environmental factors and species on yield (Figure 7b). To prevent the model from overfitting to the limited training dataset, tenfold cross-validation was performed. The model predictions revealed that yield was primarily influenced by environmental factors such as nitrogenase, ammonium nitrogen, DON, and C:N ratio, as well as species including *Mesorhizobium* and *Opitutus*.

### 2.7. KEGG Analysis of Nitrogen Metabolism Gene Families and Pathways in the Rhizosphere

**Figure 8:**
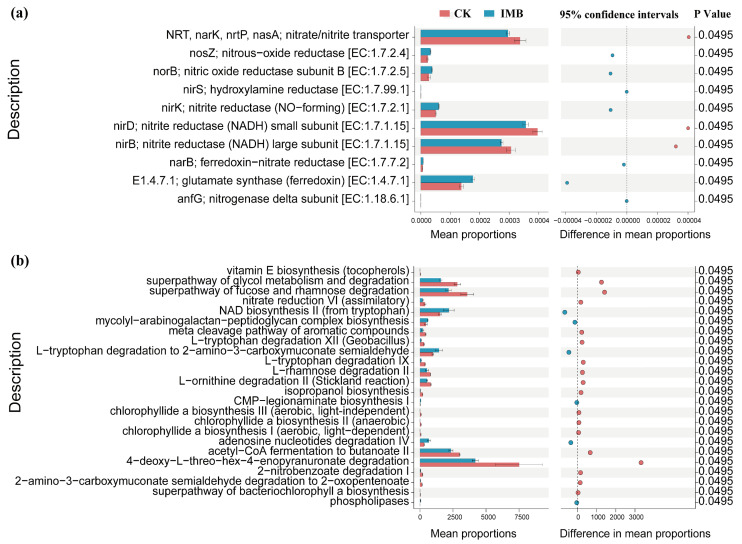
Analysis of the abundance of functional gene families and pathways of bacteria and fungi in the rhizosphere. (**a**) Differential analysis of the abundance of gene families and functional pathways related to nitrogen metabolism. (**b**) Differential analysis of the abundance of fungal metabolic pathways. CK and IMB denote the conventional level of inorganic fertilizer (control) and 30% reduction in inorganic fertilizer mixed with biochar and manure, respectively.

To further reveal the effects of biochar and composted manure on nitrogen metabolism-related signaling pathways in the rhizosphere of border rows, STAMP was used to conduct differential analysis of their pathways. Compared to CK, the synergistic effect of biochar and mature manure significantly reduced the relative abundance of functional gene families involved in the synthesis of (nitrite) nitrate transporter proteins (NRT, nark, nrtP, nasA), as well as the small (nirD) and large (nirB) subunits of NADH-nitrite reductase (*p *< 0.05; Figure 8a). It significantly increased the relative abundance of functional genes involved in the synthesis of nitrous oxide reductase (nosZ), nitric oxide reductase (norB), hydroxylamine reductase (nirS), ferredoxin-nitrite reductase (narB), ferredoxin-glutamate synthase, (dissimilatory) nitrite reductase (nirK), and nitrogenase (anfG) (*p *< 0.05; Figure 8a). Additionally, the synergistic effect of biochar and composted manure significantly reduced the relative abundance of functional pathways related to sugar metabolism and degradation, as well as the synthesis of amino acids and aromatic compounds (*p *< 0.05; Figure 8b), while significantly increasing the relative abundance of functional pathways related to lipid synthesis (*p *< 0.05; Figure 8b). The above results indicate that under low-fertilizer conditions, biochar and composted manure significantly enhanced the pathways of rhizosphere nitrogen fixation, anaerobic ammonia assimilation, and denitrification at the border, while reducing the pathways of dissimilatory (assimilatory) nitrate reduction and nitrification (Figure S5).

## 3. Discussion

### 3.1. Biochar and Composted Manure Synergistically Regulated Rhizosphere Nitrogen Dynamics to Enhance Maize Yield

In this study, reducing inorganic fertilizer led to a significant decrease in maize yield and land use efficiency within the intercropping system (Figure 1), while the organic nitrogen content in the rhizosphere of border rows significantly increased (Figure 2b). Concurrently, there was a notable increase in the contents of nitrate reductase and nitrogenase (Figure 3b). This could be attributed to the fact that when nitrogen nutrients in the rhizosphere of border rows decreased, the roots of the intercropped plants reduced their reliance on exogenous organic nitrogen. Instead, they enhanced nitrogen reduction and nitrogen fixation by increasing the activities of nitrate reductase and nitrogenase, thereby optimizing nitrogen utilization [23,24]. However, this efficiency is relatively low and poses a significant risk of nitrogen loss, leading to reduced maize yields. To address this issue, under the reduction conditions, the application of biochar or composted manure compensated for the yield loss caused by the reduction (Figure 1), and the greatest increase in yield was observed when both were applied together.

In complex intercropping systems, the increase in maize yield is attributed to multiple factors. Dong et al. [25] found that the yield of maize crops in the maize and peanut intercropping system increased with the rise in ammonium nitrogen content. Zhang et al. [26] also discovered in pot experiments that maize roots exhibited a significantly higher absorption and growth preference for ammonium nitrogen compared to nitrate nitrogen. In fact, the preferred nitrogen form varies among the same crop roots across different environments and genotypes [27,28]. In this study, the increase in maize yield was significantly correlated with the synergistic enhancement of ammonium nitrogen concentration in the rhizosphere of border rows by biochar and composted manure. On the one hand, the herbaceous biochar (maize straw) used in this experiment exhibits higher adsorption capacity compared to woody biochar [29]. The acidic functional groups (such as carboxyl groups) on its surface can electrostatically adsorb NH_4_^+^ [30], thereby promoting the fixation of ammonium nitrogen in the rhizosphere and thus achieving yield enhancement. Furthermore, Sha et al. [31] found that the mixture of biochar and organic fertilizer can reduce ammonia volatilization, but combining it with amino fertilizers increased ammonia volatilization. Yan et al. [32] also discovered that the co-application of biochar and manure promoted the transformation of inert nitrogen into active nitrogen, thereby increasing the nitrogen uptake rate to a certain extent and achieving yield growth, which is consistent with the findings of this study. On the other hand, although intercropping enhances interspecific root interactions and development [33], this complementary relationship may quickly turn into competition [34]. However, the adsorption properties of biochar and the organic matter residues from manure generate a substantial amount of rhizospheric nitrogen deposition. Simultaneously, the porosity of biochar increases the root space, which avoids interspecific competition in intercropping. This enables maize roots to effectively acquire part of the fixed ammonium nitrogen produced by the symbiotic nitrogen fixation of soybean roots [23], thereby achieving an increase in production.

In addition, the yield of maize crops is significantly positively correlated with the content of particulate organic nitrogen and soluble organic nitrogen in the rhizosphere of border rows and significantly negatively correlated with the content of mineral-associated organic nitrogen (Figure 7a). A possible explanation is that the collaboration of biochar and composted manure significantly increased the TC content in the roots of border rows (Table S4). This enhancement promoted rhizosphere carbon deposition, induced the rhizosphere priming effect, and subsequently enhanced the mineralization of mineral-associated organic nitrogen [35], ultimately leading to increased corn yield. Furthermore, Zhang et al. [36] found that biochar increased the retention of particulate organic nitrogen and total mineral-associated nitrogen in fertilizer nitrogen. Additionally, Qu et al. [37] found that the rhizosphere C:N ratio was significantly positively correlated with the content of mineral-associated organic nitrogen and particulate organic nitrogen. This aligns with the results of this study; i.e., a higher rhizosphere soil C:N ratio can enhance nitrogen fixation and the storage of organic nitrogen [38,39], thereby consistently improving the yield of intercropped maize crops.

### 3.2. Biochar and Mature Manure Synergistically Altered Keystone Rare Species Communities to Regulate Rhizosphere Nitrogen Dynamics

To further explore the mechanisms by which biochar and composted manure synergistically regulate nitrogen dynamics in the rhizosphere of border rows under low-fertilizer conditions, the focus can be placed on nitrogen-functional microbial communities that mediate nitrogen metabolism, transport, and uptake processes. Diversity analysis revealed that biochar and composted manure had no significant impact on the diversity and distribution structure of nitrogen-cycling bacterial communities, but they significantly altered the species evenness and spatial distribution characteristics of symbiotic fungal communities closely related to the nitrogen cycle (Figure 4). At the phylum level, biochar and composted manure synergistically significantly reduced the relative abundance of *Arbuscular Mycorrhizal Fungi* (*AMF*) in the rhizosphere of border rows. Elzobair et al. [40] also found that biochar and manure significantly reduced the colonization of *AMF*. This may be due to the significant preference differences exhibited by *AMF* for the rhizosphere microenvironment [20,41], while the phenolic and polyphenolic compounds present in biochar exert an inhibitory effect on the growth of *AMF* in this environment [42]. Furthermore, extensive research has demonstrated that *AMF* is closely associated with nitrogen fixation and nitrogen transfer in leguminous plants [43,44,45]. Therefore, another possible explanation is that maize crops can acquire nitrogen through the biological nitrogen fixation of soybean plants, while well-rotted manure provides a substantial amount of organic nitrogen, and the porous structure of biochar immobilizes it, reducing nitrogen loss. This may, to some extent, diminish the role of *AMF* in nutrient uptake [46], thereby reducing the colonization of *AMF* in the rhizosphere of border rows.

Furthermore, under low-fertilizer conditions, biochar and mature manure did not significantly affect high-abundance nitrogen-cycling bacterial communities, but they significantly altered low-abundance nitrogen-cycling bacterial communities. At the genus level, biochar and matured manure significantly increased the relative abundance of *Mesorhizobium*, *Opitutus*, *Nitrosospira*, *Anaerostipes*, and *Starkeya*, while significantly decreasing the relative abundance of *Klebsiella* (Figure S2a). Among these, the genus *Nitrosospira* is a type of ammonia-oxidizing bacteria (*AOB*) that oxidizes ammonia to nitrite [47]. The genus *Klebsiella* is a heterotrophic nitrifier with both nitrification and aerobic denitrification capabilities [48,49], and some strains (e.g., *roggenkampii ED5*) possess nitrogen-fixing abilities [49,50]. The genus *Mesorhizobium* can form symbiotic nodules with leguminous crops, participate in the functional tricarboxylic acid (TCA) cycle, and perform biological nitrogen fixation [51]. The genus *Opitutus* can reduce nitrate to nitrite [52]. The *YW6* strain of the *Starkeya* genus is involved in the ammonification process that converts organic nitrogen into inorganic nitrogen [53]. Biochar and mature manure selectively enriched low-abundance autotrophic nitrifying bacteria, biological nitrogen-fixing bacteria, ammonifying bacteria, and some denitrifying bacteria, while inhibiting genera with heterotrophic nitrification and aerobic denitrification capabilities. A potential reason for this phenomenon is the synergistic creation of a high C:N rhizosphere microenvironment by biochar and mature manure. Another possible explanation is that the porous structure of biochar provides physical protection for specific microorganisms, shielding them from environmental stress, while adsorbing nutrient elements from manure, thereby creating a suitable habitat that fosters their aggregation [54].

### 3.3. Biochar and Mature Manure Synergistically Altered Nitrogen Metabolic Enzymes and Pathways

To gain a deeper understanding of the mechanisms by which biochar and mature manure synergistically regulate nitrogen dynamics in the boundary-row rhizosphere under reduced fertilizer conditions, the most direct approach is to analyze changes in nitrogen-metabolizing enzymes and related pathways. In this study, the synergistic application of biochar and mature manure enhanced the activities of nitrate reductase, hydroxylamine reductase, and nitrogenase in the boundary-row rhizosphere, while significantly reducing urease activity (Figure 3b), which is consistent with the findings of [55,56,57]. Nitrogenase is primarily composed of two core proteins, the Fe protein and the MoFe protein, and is responsible for the biological fixation of atmospheric nitrogen (N_2_) into ammonia (NH_3_). It is a key enzyme in the synthesis of essential molecules for life, such as amino acids and nucleotides [58]. The trace elements Mo and B contained in biochar are important components of nitrogenase, which to some extent enhances its activity [59]. Additionally, the synergy between biochar and manure creates a high C:N ratio in the rhizosphere, stimulating the competitive advantage of nitrogen-fixing bacteria [60]. Nitrate reductase, an enzyme composed of multiple subunits, also contains Mo [61]. Hydroxylamine reductase is a multi-heme enzyme capable of catalyzing the reduction of hydroxylamine to ammonia [62]. Herrera et al. [63] found that the higher the concentration of ammonia, the lower the activity of all enzymes in the nitrate reduction system, which is contrary to the findings of this study. A possible explanation is that biochar can significantly enhance the dissimilatory nitrate reduction to ammonium (DNRA) pathway [64], which essentially activates the substrate consumption pathway of nitrate reductase, potentially leading to the upregulation of the activities of both nitrate reductase and hydroxylamine reductase. Urease is a crucial extracellular enzyme that catalyzes the hydrolysis of urea into ammonia, playing a significant role in the synthesis of nitrogen-containing compounds such as amino acids, proteins, and nucleic acids [65]. Biochar, characterized by its high specific surface area and abundant porous structure, can strongly adsorb urea molecules, thereby reducing their contact with urease and consequently inhibiting urease activity [66]. Another explanation is that the synergistic effect of biochar and manure increases the concentration of ammonium nitrogen and soluble organic nitrogen, leading to the suppression of urease synthesis [67].

Nitrogen metabolic enzymes are not only influenced by metabolic intermediates or end products but also stringently regulated at the metabolic pathway level [68,69]. This study confirms that under reduced conditions, the application of biochar and matured manure decreased the relative abundance of functional pathways related to sugar metabolism and degradation, as well as the synthesis of amino acids and aromatic compounds, while significantly increasing the relative abundance of the functional pathway for lipid synthesis (Figure 8b), which is consistent with the findings of other studies [70]. Amino acids are the building blocks of proteins and are also a critical link in nitrogen metabolism [71]. The research of Hu et al. [72] has shown that the synthesis and transformation of amino acids are primarily controlled by soil C and N sources. Therefore, the high C:N rhizosphere microenvironment created by the synergy of biochar and compost prompts microorganisms to preferentially decompose and utilize amino acids as a nitrogen source rather than synthesizing them, thereby inhibiting the synthesis of amino acids. Additionally, NH_4_^+^ is a direct nitrogen source for the efficient synthesis of amino acids by microorganisms [72]. Biochar reduces the concentration of amino acids available to microorganisms in the soil solution by adsorbing NH_4_^+^ and amino acids, thereby limiting microbial uptake of amino acids and consequently reducing the microbial synthesis of amino acids. Sugar metabolism and degradation are the core processes by which microorganisms acquire energy and carbon sources, while lipids serve as crucial energy storage forms and cell membrane components [73]. The reduction in sugar metabolism and the increase in lipid synthesis can alter the carbon and nitrogen utilization strategies and energy allocation patterns of soil microbial communities [74]. Such changes are also attributed to the addition of biochar and manure, which modify the competition and utilization efficiency of microorganisms for carbon and nitrogen sources. Beyond the roles of biochar and manure, intercropping systems themselves have developed multiple strategies to regulate the transformation and uptake of different nitrogen forms within the rhizosphere nitrogen pool. For example, maize root exudates, such as strigolactones and flavonoids, consistently enhance nodule renewal and biomass in leguminous plants [25,75]. In exchange, legumes under nitrogen-limited conditions fix atmospheric N_2_ via symbiosis and transfer a portion of the fixed nitrogen to non-legumes [23,24], while also depositing residual nitrogen in the rhizosphere. This rhizosphere nitrogen deposition, combined with complementary nitrogen supply strategies, is enhanced by the effects of biochar and manure. It not only enriches the soil nitrogen pool but also increases the amount of nitrogen available to plants.

## 4. Materials and Methods

### 4.1. Experiment Design

The field experiment was conducted from May 2024 to October 2024 at the Xiangyang Experimental Station of Northeast Agricultural University, located in Xiangfang District, Harbin City, Heilongjiang Province, China (45°34′–45°46′ N, 126°22′–126°50′ E). The site experiences a cold temperate continental monsoon climate characterized by rain and heat in the same season. The frost-free period lasts approximately 140 days, with ≥10 °C accumulated temperature of approximately 2700 °C. Precipitation (400–600 mm) primarily occurs during July and August each year. The soil type is mollisol, with the following fundamental physical properties: pH 6.05, bulk density 1.72 g/cm^3^, conductivity 92.45 µS/cm, total nitrogen 1.27 g/kg, total phosphorus 0.37 g/kg, total potassium 18.98 g/kg, organic carbon 14.18 g/kg.

**Figure 9:**
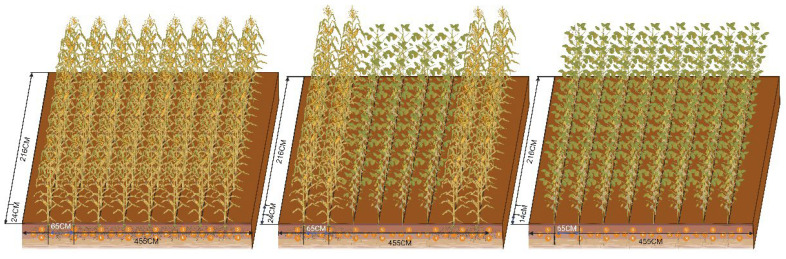
Experimental plots for intercropping maize and soybeans (created in BioRender. RPH, r. (2025); https://BioRender.com/g04r359 (accessed on 24 November 2025)).

The experiment was designed as a completely randomized block design, with five treatments: 750 kg/ha^−1^ inorganic compound fertilizer (CK), 525 kg/ha^−1^ inorganic compound fertilizer (I), 525 kg/ha^−1^ inorganic compound fertilizer and 7500 kg/ha^−1^ manure (IM), 525 kg/ha^−1^ inorganic compound fertilizer and 10 t/ha^−1^ biochar (IB), 525 kg/ha^−1^ inorganic compound fertilizer, 7500 kg/ha^−1^ manure, and 10 t/ha^−1^ biochar (IMB). Each treatment had 3 replicates, and the compound fertilizer had a nutrient formulation of 12-18-15 (N-P_2_O_5_-K_2_O). All treatments mentioned above refer to the fertilizer application rates for the corn plots, while the fertilizer application rates for the soybean plots are shown in Table S2. The size of each plot was 4.55 m × 2.16 m. A 1.3 m buffer zone was set up between adjacent plots to minimize cross-interference, and the detailed field layout within each plot is illustrated in Figure 9. 

### 4.2. Manure and Biochar

The experimental manure was purchased from Hebei Daoqin Biotechnology Co., Ltd. (Shijiazhuang, China) The raw material consists of fully naturally decomposed pig manure. Its chemical properties were as follows: pH 7.5, organic matter 77.4%, moisture content 13.2%. The biochar for the experiment was purchased from Liaoning Jinhefu Development Co. Ltd. The raw material was corn stover, which was burned at 450 °C under anaerobic conditions, and its physical and chemical properties were as follows: powder form, bulk density 0.4 g/cm^3^, specific surface area 84.3 m^2^/g, conductivity 1.2 dS/m, pH 8.75, organic matter 32.91 g/kg, total nitrogen, 1.82 g/kg, available phosphorus 29.87 mg/kg, available potassium 38.47 mg/kg, and available nitrogen 71.23 mg/kg. The biochar and manure purchased were applied without any special treatment, strictly following local farmers’ fertilization practices. They were evenly spread over the soil surface in sequence, then thoroughly mixed with the topsoil (0–20 cm layer) through repeated tilling with a rotary tiller. Two weeks later, the inorganic fertilizer was applied as a single basal application prior to sowing.

### 4.3. Soil Sampling Collection and Analysis

The soil samples were collected in September 2024, coinciding with the maize filling stage and the soybean bulking stage. In this study, we focused on the rhizosphere soil of maize plants from the border rows, which are defined as the maize rows immediately adjacent to soybean rows in the intercropping system. Five healthy plants were randomly selected in the border rows; the soil attached around the 10 cm root system was first removed with a knife, and then 1–3 cm of soil attached to the root surface (rhizosphere) was brushed. Subsequently, the soil samples were passed through a 2 mm sieve to remove roots and stones and thoroughly mixed. Mixed samples were placed in sterile self-sealing bags, transferred to the laboratory on ice, and divided into three subsamples. One subsample was stored at −80 °C for soil microbial DNA extraction; a second subsample was kept at 4 °C for analyzing enzyme activity, ammoniacal nitrogen (NH_4_^+^-N), nitrate nitrogen (NO_3_^−^-N), dissolved organic nitrogen (DON), and dissolved total nitrogen (DTN); and a third subsample was air-dried for determination of total carbon (TC), total nitrogen (TN), mineral-associated organic nitrogen (MAON), and particulate organic nitrogen (PON).

### 4.4. Soil Nutrients and Yield Measurement

At the point of maize maturity, five healthy plants were randomly selected from border rows in order to measure grain yield. Grain samples were subjected to oven-drying and subsequent weighing, and the final yield was then adjusted to 14% moisture content for maize and 13% for soybeans. The soil sample was weighed, and the nitrogenase (Nase) activity was measured using the NITS ELISA kit (product no. A-K00427B, Jiangsu Jingmei Biotechnology Co., Ltd., Yancheng, China) on a microplate reader. Subsequent enzymatic analyses included hydroxylamine reductase (HR, Product No. G03334W), urease (Ure, Product No. G0301W), and nitrate reductase (NR, Product No. G0309W) using corresponding kits (Grace Biotechnology Co., Ltd., Suzhou, China) with 48-well microplates. NH_4_^+^-N and NO_3_^−^-N were quantified by indophenol blue and KCl-extraction dual-wavelength methods, respectively, with DTN analyzed via a TOC analyzer, and the DON was determined by subtracting the dissolved inorganic nitrogen (NO_3_^−^-N and NH_4_^+^-N) from the DTN [76]. Following the process of air-drying the soil samples, elemental analysis (EA) was conducted for the purpose of determining the TC and TN. The extraction of PON and MAON was achieved through the use of sodium hexametaphosphate, followed by quantification via Kjeldahl nitrogen determination.

### 4.5. Soil DNA Extraction and PCR Amplification

According to the protocol provided by the manufacturer, soil samples were preserved in dry ice and transported to Hangzhou Lianchuan Biotechnology Co. (Hangzhou, China) for soil DNA extraction, PCR amplification, and sequencing. The target regions were the V3–V4 region of the 16S rRNA gene and the ITS2 region of the fungal Internal Transcribed Spacer (ITS) gene, respectively. The cetyltrimethylammonium bromide (CTAB) method was selected for the total microbiome DNA extraction from the samples, and the DNA concentration and purity were detected by means of electrophoresis in 2% agarose gel. 16S rRNA and ITS genes were amplified using specific primers (341F (5′-CCTACGGGGNGGCWGCAG-3′)/805R (5′-GACTACHVGGGTATCTAATCC-3′) and (fITS7 (5′-GTGARTCATCGAATCTTTG-3′)/ITS4 (5′-TCCTCCGCTTATTGATATATGC-3′)). In the present study, all polymerase chain reactions (PCR) comprised 12.5 µL of Phusion^®^ Hot Start Flex 2X Master Mix (NEB, M0536L), 2.5 µL of forward and reverse primers, 50 ng of DNA template, and 25 µL of ddH_2_O. The thermal cycling procedure entailed a preliminary denaturation step at 98 °C for 30 s, followed by 32 cycles of denaturation at 98 °C for 10 s, annealing at 50 °C for 30 s, and extension at 72 °C for 45 s. Thereafter, the samples were subjected to an incubation period at 72 °C for a duration of 10 min. Finally, the 1× Tae buffer was mixed with the PCR products, which were detected electrophoretically in 2% agarose gel. The PCR products were then detected using AMPure XT beads (Beckman Coulter Genomics, Danvers, MA, USA) for purification and a Qubit dsDNA HS Assay Kit (Invitrogen, Carlsbad, CA, USA) for quantification.

### 4.6. Library Generation and Illumina NovaSeq Sequencing

Sequencing libraries were generated using the NEB Next^®^ Ultra DNA Library Prep Kit (Illumina, San Diego, CA, USA) following the manufacturer’s recommendations, and index codes were added. The library quality was assessed on the Agilent 2100 Bioanalyzer (Agilent, Santa Clara, CA, USA) and using a library quantification kit from Illumina (Kapa Biosciences, Woburn, MA, USA). Finally, the library was sequenced on an Illumina NovaSeq 6000 (PE250) platform, and 250 bp paired-end reads were generated. The raw data obtained from sequencing were spliced and filtered to obtain clean data. The samples were split into data, and joints and barcode sequences were removed based on barcode information. Paired ends were merged using FLASH software (v1.2.8). The raw data were quality-controlled under specific filtering conditions to obtain high-quality clean tags based on fqtrim (v0.94). Chimeric sequences were filtered using Vsearch online software (v2.3.4). Demodulation and denoising were performed, and amplicon sequence variant (ASV) signature sequences and abundance tables were obtained using the DADA2 program. The ASV sequence files were utilized to annotate the species of bacteria and fungi using SILVA (Release138) and the RDP (unite) database, respectively.

As demonstrated in the KEGG database (map00910), nine nitrogen-cycling pathways have been identified: nitrogen fixation, nitrification, denitrification, assimilatory nitrate reduction, allochthonous nitrate reduction, nitrogen mineralization, ammonia assimilation, (sub)nitrate transport, and respiration. “FAPROTAX” annotated bacterial nitrogen cycling functions [77]. The “FUNGuildR” tool was employed for fungal functional annotation, retaining only those with a confidence level of ‘Highly Probable’ to enhance prediction accuracy [78]. Additionally, the “PICRUSt2” tool was used to predict the nitrogen cycle functional pathway (ko00910) of potential KEGG orthologs in the microbial community.

### 4.7. Data Processing and Analysis

The following statistical analyses were all conducted using the R software tool (version 4.4.1). Using the “agricolae” package, one-way ANOVA and LSD post hoc tests were conducted on the nitrogen components, nitrogen-converting enzymes, and microbial α-diversity data of the rhizosphere in boundary rows. The “FactoMineR” package was employed to perform principal component analysis (PCA) on the soil nitrogen component data. Subsequently, the “piecewiseSEM” package was employed to construct a piecewise structural equation model (SEM) with the soil nitrogen data, which was used to analyze the regression relationships among nitrogen components. The “vegan” package was employed to perform principal coordinate analysis (PCoA) based on Jaccard distance on the microbial community structure, along with PERMANOVA and one-way ANOVA. The “microeco” package was used to sequentially apply the Kruskal–Wallis test and Wilcoxon test on the microbial relative abundance data to screen for differential species, followed by the use of LefSe (LDA > 3.0) to identify biomarkers, while Chao1 and Shannon indices were calculated to represent species richness [79] and evenness [80], respectively. The microbial co-occurrence network was constructed using the “igraph” package, and the network topology and cohesion were calculated. Meanwhile, random networks were generated using the Barabasi–Albert model [81] to evaluate network cohesion. Node attributes were resampled via the bootstrapping method, and the Kolmogorov–Smirnov test was employed to examine the significance between specific networks. Finally, network visualization was performed using Gephi v0.10.1 software. The map00910 nitrogen metabolism pathway was plotted using the R package “pathview” (v1.46.0) [82,83]. Random forest analysis was performed using “randomForest”, and the significance of each predictor was assessed by using the “rfPermute” packages.

## 5. Conclusions

On the basis of reducing fertilizer application, the addition of biochar and composted manure not only compensated for the yield loss of maize caused by fertilizer reduction but also achieved a yield increase. Biochar and composted manure reduced the dependence on microbial-mediated mineralization of exogenous urea nitrogen but enhanced the processes of nitrogen fixation and denitrification, thereby promoting the accumulation of ammonium nitrogen and available organic nitrogen and achieving an increase in maize yield. At the microbial level, biochar and composted manure significantly altered the species evenness and structural characteristics of fungal communities but did not have a significant impact on the species composition or structural characteristics of bacterial communities. Specifically, biochar and composted manure reduced the relative abundance of arbuscular mycorrhizal fungi while triggering niche responses of low-abundance key microbial taxa involved in nitrogen cycling in the soil, altering nitrogen transformation processes in the border-row rhizosphere by adjusting their niches. In addition, biochar and composted manure increased the number of edges, nodes, and average degree in the nitrogen-cycling bacterial network, leading to more community participation but reducing the proportion of positive links and increasing competition. In the future, it is necessary to further explore the feedback relationships among soil, roots, and microorganisms in the border-row rhizosphere under the synergistic effects of biochar and composted manure from the perspectives of soil genomics and metabolomics.

## Data Availability

The datasets used or analyzed during the current study are available from the corresponding author upon reasonable request. The data are not publicly available due to [ongoing further analysis of the raw sequencing data, which will be deposited in the NCBI public database in a future publication].

## Supplementary Materials

The following supporting information can be downloaded at https://www.mdpi.com/article/10.3390/plants14243696/s1: Figure S1: Analysis of the relative abundance at the phylum level of rhizosphere microorganisms; Figure S2: LefSe analysis of the rhizosphere bacterial community for screening biomarkers; Figure S3: LefSe analysis of the rhizosphere fungal community for screening biomarkers; Figure S4: Random forest prediction of core species in the border-row rhizosphere; Figure S5: Nitrogen metabolic pathway diagram; Figure S6: Experimental area for intercropping maize and soybeans; Table S1: Physicochemical properties of biochar, manure, and soil (0–20 cm) in the experimental area; Table S2: Fertilizer usage table; Table S3: One-way ANOVA with an LSD test of nitrogen content and maize yield in the border-row rhizospheric samples; Table S4: One-way ANOVA with an LSD test of enzyme activity in the border-row rhizospheric samples; Table S5: One-way ANOVA with an LSD test of total bacteria and fungi in the border-row rhizospheric samples; Table S6: One-way ANOVA with an LSD test of N-cycling bacteria and symbiotic fungi in the border-row rhizospheric samples; Table S7: The topological characteristics of the co-occurrence network and random network of the N-cycling bacterial community in the border-row rhizosphere; Table S8: The topological characteristics of the co-occurrence network and random network of the symbiotic fungi community in the border-row rhizosphere; Table S9: The attributes of nodes in the collinear network of rhizosphere microorganisms were tested by the Kolmogorov–Smirnov test.

## Abbreviations

The following abbreviations are used in this manuscript:

MAON	Mineral-associated organic nitrogen
DON	Linear dichroism dissolved organic nitrogen
PON	Particulate organic nitrogen
DTN	Dissolved total nitrogen
HR	Hydroxylamine reductase
NR	Nitrate reductase
Nase	Nitrogenase
Ure	Urease
