# Supplementary material for: Effect of Biochar and Well-Rotted Manure on Maize Yield in Intercropping Systems Based on High-Throughput Sequencing Technology
Source: Plants (Basel). 2025 Dec 5;14(24):3696. doi: 10.3390/plants14243696 (PMC12736783; doi:10.3390/plants14243696)
Supplement: Supplementary file 1 [file plants-14-03696-s001.zip › plants-3858121-supplementary.pdf]

Table S1. Physicochemical properties of biochar, manure, and soil (0-20 cm) in the experimental area.

Properties of biochar	Value	Properties of manure	Value	Properties of soil	Value
Materials	Maize straw	Moisture (%)	13.2	pH	6.05
Process parameters	450℃; Anaerobic	OM (%)	77.4	Conductivity (μs/cm)	92.45
pH	8.75	pH	7.5	TN (g/kg)	1.27
BD (g/cm^3)	0.40	FC (CFU/g)	≤100	TP (g/kg)	0.37
SOM (g/kg)	32.91	Pb (mg/kg)	22	TK (g/kg)	18.98
TN (g/kg)	1.82	Cd (mg/kg)	-	BD (g/cm^3)	1.72
AK(mg/kg)	38.47	Cr (mg/kg)	8	SOC (g/kg)	14.18
AP (mg/kg)	29.87	As (mg/kg)	5		
AN (mg/kg)	71.23	Hg (mg/kg)	0.4		

Notes: pH denotes the potential of hydrogen; BD denotes bulk density; SOM denotes soil organic matter; OM denotes organic matter; FC denotes fecal coliform count; TN, TP, and TK denote total nitrogen, total phosphorus, and total potassium, respectively; AK, AP, and AN denote available potassium, available phosphorus, and available nitrogen, respectively; Pb, Cd, Cr, As, and Hg denote lead, cadmium, chromium, arsenic, and mercury, respectively; SOC denotes soil organic carbon.

Table S2. Fertilizer usage table.

**Treatment**	**Fertilizer dosage**	**Specific information (kg/ha)**
CK	Only apply inorganic fertilizers	M:750; S:450
I	Apply 70% inorganic fertilizer	M:525; S:315
IM	Apply 70% inorganic fertilizer mixed with pig manure	M:525 inorganic fertilizers, 7500 pig manure; S:315 inorganic fertilizers, 4500 pig manure
IB	Apply 70% inorganic fertilizer mixed with biochar	M:525 inorganic fertilizers, 10,000 biochar; S:315 inorganic fertilizers, 10,000 biochar
IMB	Apply 70% inorganic fertilizer mixed with biochar and manure	M:525 inorganic fertilizers, 7500 pig manure, 10,000 biochar; S:315 inorganic fertilizers, 4500 pig manure, 10,000 biochar

Notes: S and M denote soybean (Heilong 84 variety) and maize (Yibang 2 variety), respectively; inorganic fertilizer was formulated as N-P_2O_5-K_2O 12-18-15.

Table S3. One-way ANOVA with an LSD test of nitrogen content and maize yield in the border-row rhizospheric samples.

**Treatment**	**TN %**	**PON g/kg**	**DON mg/kg**	**NH_4^+-N mg/kg**	**NO_3^--N mg/kg**	**MAON g/kg**	**DTN mg/kg**	**Yield kg/ha**
CK	0.14±0.00^b	0.15±0.03^c	0.98±0.24^b	0.85±0.03^{bc}	12.03±0.90^a	1.25±0.03^a	13.87±0.10^b	5098.57±413.69^c
I	0.14±0.02^b	0.30±0.04^{bc}	6.76±4.45^a	0.49±0.18^c	9.81±6.44^{ab}	1.07±0.19^a	17.1±1.91^a	3515.53±273.66^d
IM	0.15±0.01^b	0.52±0.16^{bc}	3.37±1.30^{ab}	0.42±0.14^c	9.36±1.34^{ab}	0.95±0.25^{ab}	13.2±1.52^b	6558.93±551.68^b
IB	0.15±0.02^b	0.69±0.07^b	4.48±1.24^{ab}	1.04±0.11^{ab}	4.90±0.73^b	0.84±0.26^{ab}	10.4±0.46^c	7476.28±455.90^a
IMB	0.19±0.03^a	1.40±0.48^a	6.11±0.99^a	1.32±0.51^a	7.47±2.41^{ab}	0.5±0.40^b	14.9±1.82^{ab}	7849.44±318.92^a

Notes: TN denotes total nitrogen; PON, DON, and MAON denote particulate organic nitrogen, dissolved organic nitrogen, and mineral-associated organic nitrogen, respectively; NH_4^+-N and NO_3^--N denote ammonium nitrogen and nitrate nitrogen, respectively; DTN denotes dissolved total nitrogen.

Table S4. One-way ANOVA with an LSD test of enzyme activity in the border-row rhizospheric samples.

Treatment	Hydroxylamine Reductase μg/d/g	Nitrate Reductase nmol/d/g	Urease μg/d/g	Nitrogenase IU/g	TC %
CK	162.62±2.39^{d}	6.55±0.11^{e}	897.33±39.90^{a}	1.42±0.04^{c}	1.57±0.34^{b}
I	160.20±0.72^{e}	15.16±1.46^{d}	753.73±44.69^{b}	1.69±0.03^{a}	1.83±0.34^{b}
IM	169.45±0.59^{c}	20.80±1.10^{c}	675.61±18.16^{c}	1.51±0.02^{b}	1.90±0.34^{b}
IB	187.95±1.39^{b}	44.15±2.01^{b}	846.91±51.61^{a}	1.38±0.03^{c}	2.75±0.34^{ab}
IMB	215.45±0.32^{a}	103.98±2.31^{a}	719.80±35.99^{bc}	1.55±0.03^{b}	3.80±0.34^{a}

Notes: TC denotes total carbon.

Table S5. One-way ANOVA with an LSD test of total bacteria and fungi in the border-row rhizospheric samples.

Treatment	Total bacteria		Total fungi	
	Shannon	Chao1	Shannon	Chao1
CK	10.70±0.15^{a}	3275.53±199.28^{a}	6.55±0.24^{ab}	466.98±23.96^{b}
I	10.78±0.04^{a}	3406.42±51.40^{a}	5.92±0.57^{b}	461.31±21.50^{b}
IM	10.69±0.16^{a}	3345.14±272.02^{a}	5.84±0.44^{b}	464.38±30.02^{b}
IB	10.75±0.23^{a}	3213.72±304.93^{a}	6.65±0.33^{a}	539.14±20.93^{a}
IMB	10.85±0.07^{a}	3548.49±231.12^{a}	6.46±0.24^{ab}	520.32±13.52^{a}

Table S6. One-way ANOVA with an LSD test of N-cycling bacteria and symbiotic fungi in the border-row rhizospheric samples.

Treatment	N-cycling bacteria*		Symbiotic fungi	
	Shannon	Chao1	Shannon	Chao1
CK	3.86±0.13^{a}	100.67±12.05^{a}	1.87±0.14^{a}	24.00±3.61^{ab}
I	4.05±0.11^{a}	106.33±7.77^{a}	1.86±0.77^{a}	24.33±6.81^{a}
IM	4.08±0.11^{a}	112.33±18.48^{a}	1.84±0.53^{a}	22.67±4.73^{ab}
IB	3.96±0.05^{a}	103.33±8.14^{a}	1.15±0.03^{ab}	25.33±2.08^{a}
IMB	4.02±0.18^{a}	116.33±16.77^{a}	0.90±0.20^{b}	16.67±1.16^{b}

Note: N-cycling bacteria*, where N denotes nitrogen.

Table S7. The topological characteristics of the co-occurrence network and the random network of the N-cycling bacterial community in the border-row rhizosphere.

Treatment	Empirical networks						Random networks	
	Nodes	Edges	TRP	AD	ACC	APL	ACC	APL
CK	198	3595	89%	36.31	1.00	1.00	0.288±0.023	1.836±0.026
IB	211	4531	88%	42.95	1.00	1.00	0.307±0.022	1.814±0.026
IM	216	4375	89%	40.51	1.00	1.00	0.293±0.022	1.829±0.026
IMB	226	5030	87%	44.51	1.00	1.00	0.303±0.022	1.817±0.026

Notes: TRP denotes the ratio of positive links; AD denotes the average degree; ACC denotes the average clustering coefficient; APL denotes the average path length.

Table S8. The topological characteristics of the co-occurrence network and random network of the symbiotic fungi community in the border-row rhizosphere.

Treatment	Empirical networks						Random networks	
	Nodes	Edges	TRP	AD	ACC	APL	ACC	APL
CK	49	219	97%	8.94	1.00	1.00	0.232±0.027	2.072±0.039
IB	62	427	98%	13.77	1.00	1.00	0.313±0.023	1.845±0.028
IM	50	276	97%	11.04	1.00	1.00	0.321±0.023	1.841±0.028
IMB	40	162	98%	8.10	1.00	1.00	0.266±0.027	1.982±0.036

Notes: TRP denotes the ratio of positive links; AD denotes the average degree; ACC denotes the average clustering coefficient; APL denotes the average path length.

Table S9. The attributes of nodes in the collinear network of rhizosphere microorganisms were tested by the Kolmogorov–Smirnov test.

	Comparison	Degree	Betweenness	Closeness	Transitivity	Dissimilarity of subnetworks
N-cycling bacteria*	IB vs CK	p<0.0001	p<0.0001	p<0.0001	p<0.0001	**0.986**
	IM vs CK	p<0.0001	p<0.0001	p<0.0001	p<0.0001	**0.984**
	IMB vs CK	p<0.0001	p<0.0001	p<0.0001	p<0.0001	**0.991**
Symbiotic fungi	IB vs CK	p<0.0001	p<0.0001	p<0.0001	p<0.0001	**0.979**
	IM vs CK	p<0.0001	p<0.0001	p<0.0001	p<0.0001	**0.996**
	IMB vs CK	p<0.0001	p<0.0001	p<0.0001	p<0.0001	**0.995**

Note: The attributes of network nodes are calculated through a guiding method with 10,000 iterations. In order to determine the differences in microbial networks among different fertilization management, the network dissimilarity index of Poisot et al. (2012) [1] was calculated for comparison. N-cycling bacteria*, where N denotes nitrogen.

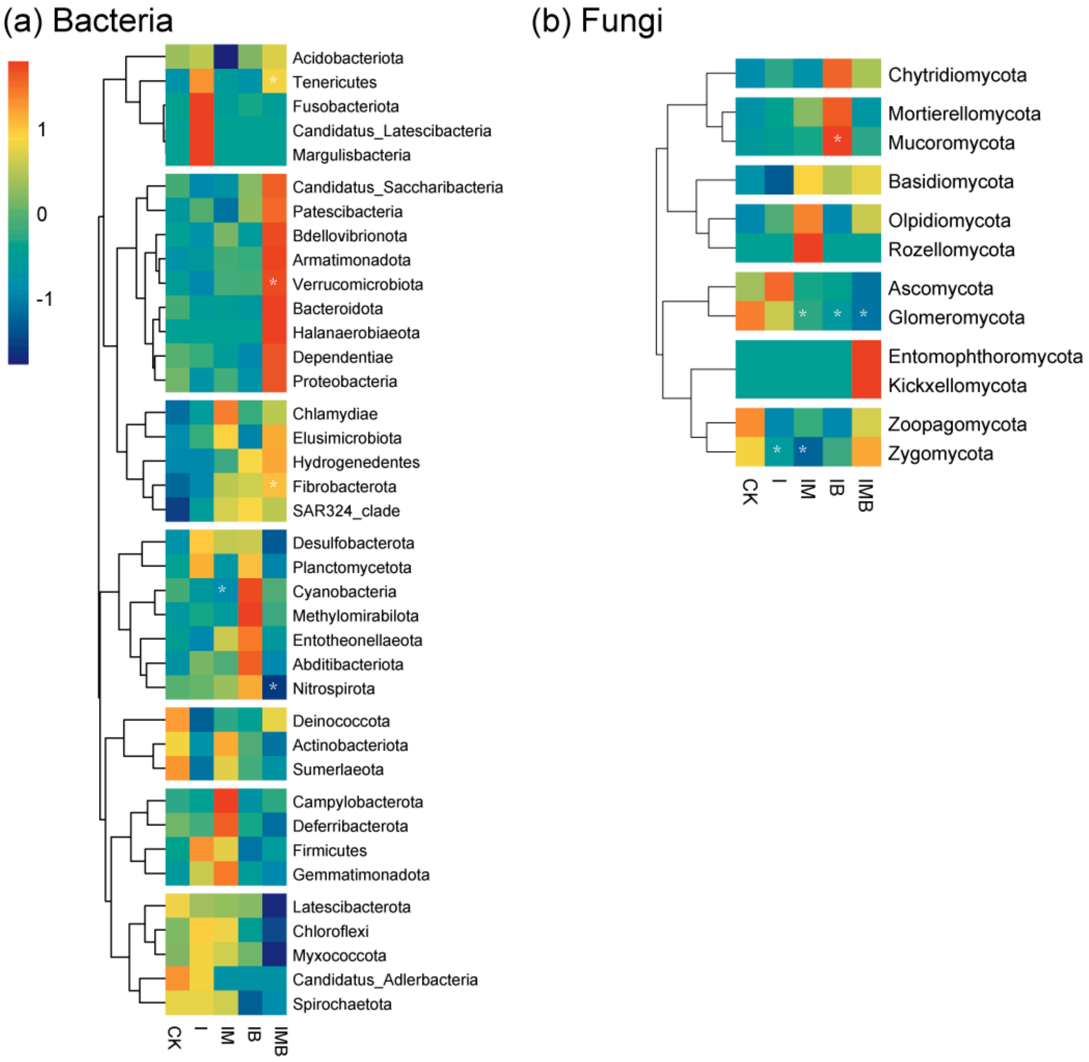

Figure S1. Analysis of the relative abundance at the phylum level of rhizosphere microorganisms. Each column of samples has undergone z-score processing, allowing for horizontal comparison. Vertical cross-species comparison is meaningless. The color represents the magnitude of the value. Warm tones indicate high values, and cool tones indicate low values.

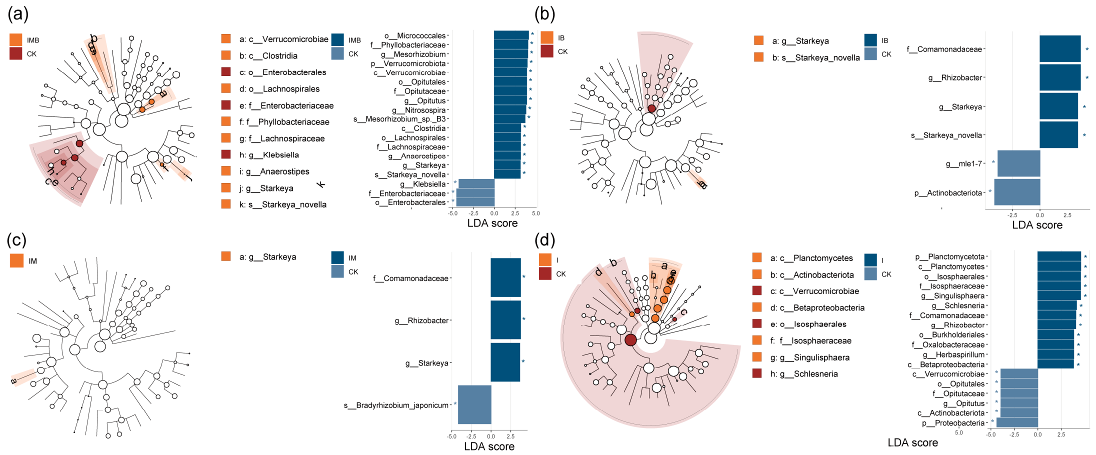

Figure S2. LefSe analysis of the rhizosphere bacterial community for screening biomarkers, LDA>3.0.

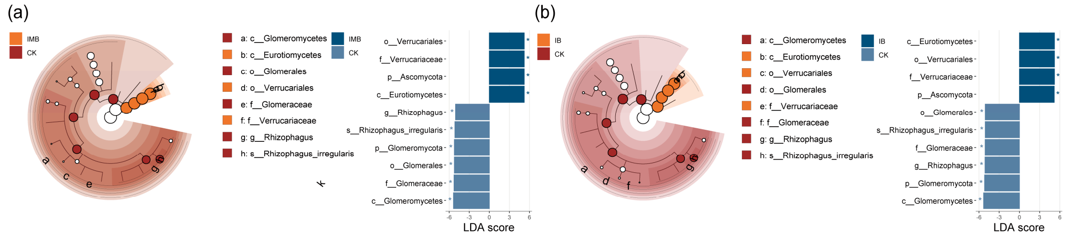

Figure S3. LefSe analysis of the rhizosphere fungal community for screening biomarkers, LDA>3.0.

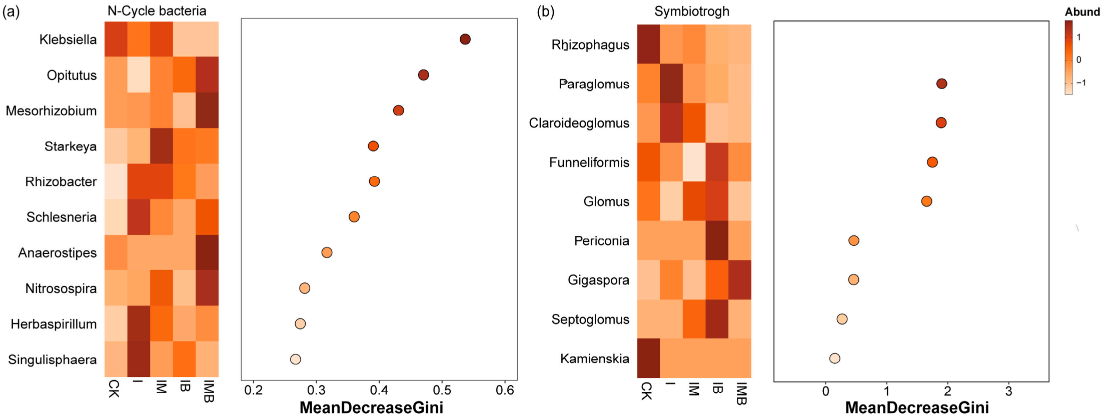

Figure S4. Random forest prediction of core species in the rhizosphere boundary line.

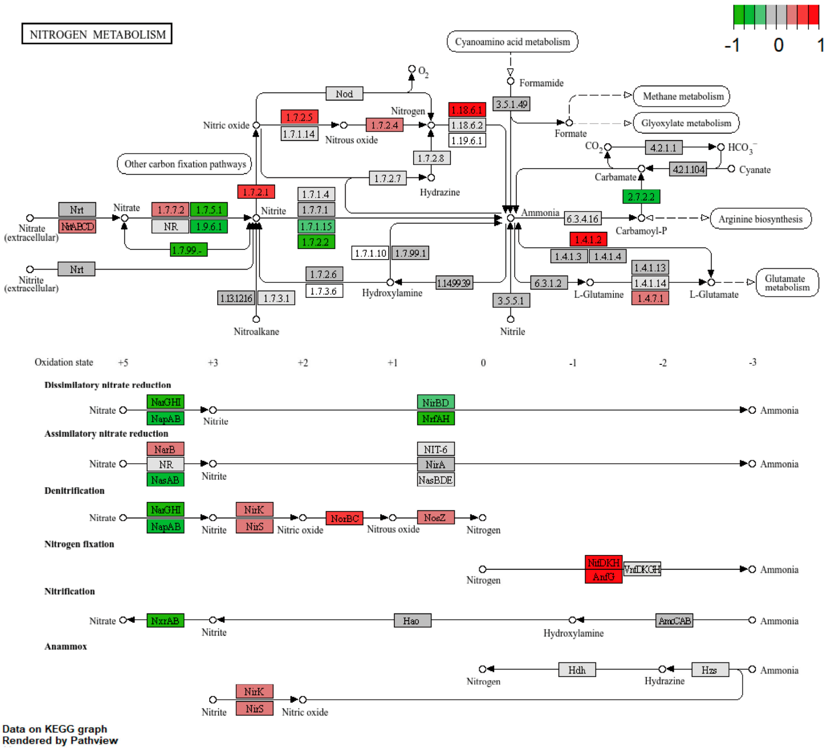

Figure S5. Nitrogen metabolic pathway diagram. The color variation represents the level of functional gene expression, with green indicating upregulation and red indicating downregulation.

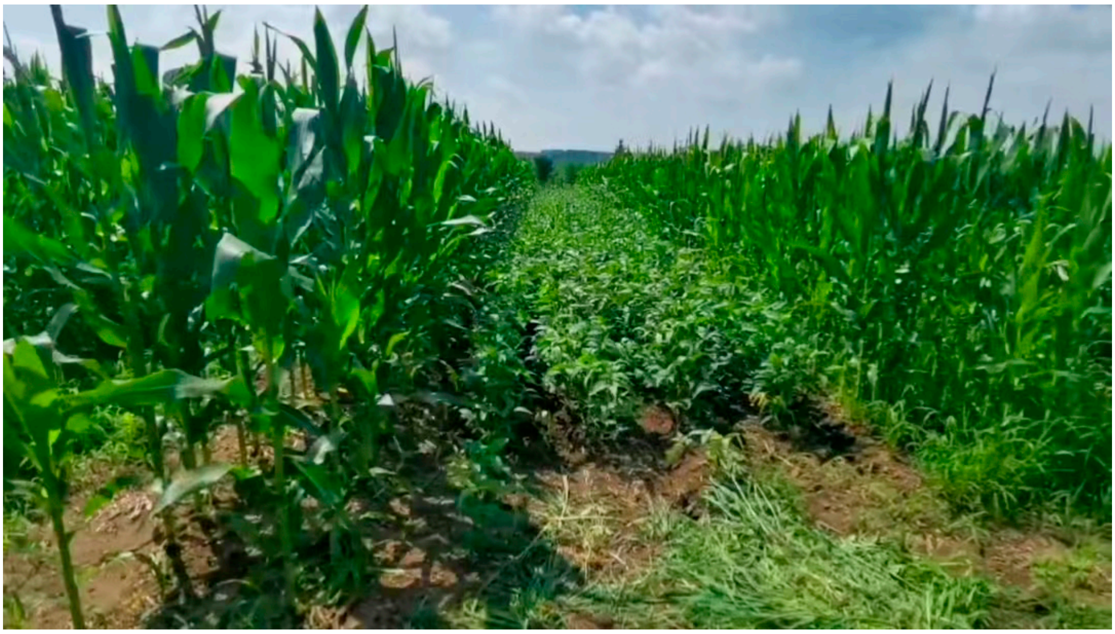

Figure S6. Experimental area for intercropping maize and soybeans.

1. Poisot, T.; Canard, E.; Mouillot, D.; Mouquet, N.; Gravel, D. The dissimilarity of species interaction networks. *Ecol. Lett.* **2012**, *15*, 1353-1361, https://doi.org/10.1111/ele.12002.
